# Supplementary material for: Are carbon emissions trading and green financial instruments synergistic? -Comprehensive quantitative research based on content analysis
Source: PLoS One. 2024 Mar 7;19(3):e0298601. doi: 10.1371/journal.pone.0298601 (PMC10919601; doi:10.1371/journal.pone.0298601)
Supplement: S1 Data — (ZIP) [file pone.0298601.s001.zip › Data/地级市Z_F工作报告.zip!/╕╩╦α/┬ñ─╧╩╨/└┤╫╘╣1⁄2╓┌║┼í╛┬φ┐╦╩2╛▌═°í┐_2017.docx]

各位代表：

  现在，我代表陇南市人民政府向大会作工作报告，请予审议，并请各位政协委员和列席人员提出意见。

  本届政府工作回顾

  2011年11月陇南市第三届人民代表大会第一次会议以来，面对复杂严峻的经济形势，在省委、省政府和市委的坚强领导下，在市人大、市政协的监督支持下，在政府全体同志的共同努力下，市政府认真贯彻党的十八大、十八届三中、四中、五中、六中全会和习近平总书记系列重要讲话精神，主动适应新常态，牢牢把握稳中求进总基调，团结带领各级政府，深化实施“433”发展战略，统筹推进稳增长、促改革、调结构、惠民生各项工作，圆满完成了本届政府确定的各项目标任务，为实现整体脱贫、与全省全国一道全面建成小康社会奠定了坚实基础。

  ———着力稳增长促发展，经济综合实力明显增强。始终把加快发展作为第一要务，抢抓政策机遇，狠抓工作落实，全市经济持续快速发展。五年来，全市GDP跨越两个百亿元台阶，2015年达到315亿元，增速居全省第1，较2011年前移了11位，年均增长10.8%；今年前三季度完成261.1亿元，增长8.2%，预计全年完成340亿元，增长8.3%。社会消费品零售总额2015年完成90.8亿元，年均增长13.3%；今年前三季度完成70.4亿元，增长9%，预计全年完成99亿元，增长9%，较2011年翻了一番。财政收入提前完成了“十二五”规划目标，收支实现了高幅增长，其中大口径财政收入2015年完成51.5亿元，年均增长19%，今年前三季度完成35.7亿元，增长3.2%，预计全年完成55.6亿元，增长8%，较2011年翻了一番；一般公共预算收入2015年完成25.5亿元，年均增长21.7%，今年前三季度完成18.5亿元，增长7.1%，预计全年完成26.5亿元，增长8%，较2011年翻了一番；一般公共预算支出2015年完成197亿元，年均增长16%，较2011年翻了近一番，今年前三季度完成149亿元，增长10.5%，预计全年完成214亿元，增长10%。农村居民人均可支配收入2015年完成5405元，年均增长14.7%，较2011年翻了一番；今年前三季度完成4351.6元，增长10.4%，预计全年完成6108元，增长13%。城镇居民人均可支配收入2015年完成18915元，年均增长11.2%；今年前三季度完成15119.4元，增长7.8%，预计全年完成20617元，增长9%。

  ———积极调结构促转型，产业质量效益不断提升。一、二、三产比例由2011年的25.6：30.6：43.8调整为2015年的22.3：23.1：54.6，产业结构更趋优化。农业经济稳步发展，“365”现代农业发展行动计划稳步推进，强农惠农政策、科技增粮措施全面落实，粮食总产实现了“九连增”。深入推进农业特色产业提质增效，每年安排农业特色产业发展基金1000万元，新增基地面积200万亩、累计达到1121万亩，完成核桃、花椒、油橄榄高接换优1045万株，中药材标准化种植、苹果矮砧密植示范基地建设进展良好，新型农业经营主体培育、农特产品品牌建设成效显著。我市被国家林业局确定为全国油橄榄示范基地，发起成立了中国油橄榄产业创新战略联盟，举办了农业特色产业扶贫国际论坛。退耕还林还草任务全面完成，土地、林地流转稳步推进，农业适度规模经营效益明显提升。连续四年成功举办农特产品产销对接洽谈会。祥宇油橄榄日处理560吨鲜果生产线、康县5万吨核桃乳生产线、礼县大黄提取液生产线建成投产，产业化经营水平进一步提升。新建标准化规模养殖场(小区)195个，生态放养鸡、中蜂养殖规模效益逐年提升，规模化养殖比重达到41%。2015年全市农业增加值完成70.3亿元，年均增长6.1%；今年前三季度完成60.3亿元，增长5.3%，预计全年增长5.5%。工业经济总量不断扩大，有色冶金、白酒酿造、农特产品加工、建筑建材、水电能源、现代制药等工业主导产业不断发展壮大，2015年全市规上工业增加值完成41.2亿元，年均增长15%；今年前三季度完成27.1亿元，增长7.1%，预计全年增长8.5%。金徽酒业优质酒技改、郭家沟铅锌矿、礼县紫金矿业、青峰石材、两当金润玉和苗家坝、橙子沟水电站等21个投资亿元以上的工业项目建成投产，累计新增规上工业企业48户；陇南经济开发区及各县区经济开发区建设取得积极进展，入园企业达到154户，较2011年新增76户；积极培育壮大中小微企业，预计今年非公经济增加值完成195.2亿元，占GDP的57.4%。第三产业快速发展，建成了陇南特色农产品交易市场、陇南电子商务产业孵化园、宕昌县哈达铺华昌药材城、陇东南苗木市场等一批物流仓储、专业市场。积极落实“互联网+”行动，淘宝网“特色中国·陇南馆”2014年开馆运营，创建了西北地区首家电商职业学院，成县、武都农村淘宝服务中心和75个村级服务站规范运营，成县、礼县、宕昌县全国电子商务进农村示范县顺利实施，阿里巴巴•陇南产业带启动上线，电子商务实现了突破性发展。加快文化旅游融合发展，宕昌县官鹅沟5A级大景区创建工作进展顺利，全市4A级景区达到9家，两当兵变纪念馆及旧址改造、文县白马人民俗综合体验区、西和乞巧文化苑等项目建成投用，“十村百户千床”示范工程取得明显成效，建成康县花桥、凤凰谷等乡村旅游示范村40个，连续成功举办乞巧女儿节、白马人民俗文化旅游节等系列节会，国家、省级非物质文化遗产申报和古建筑、古民居保护开发成效明显。官鹅沟旅游景区门票收入首次突破千万元。2015年全市文化产业增加值完成5.7亿元，年均增长26%；旅游综合收入完成48.8亿元，年均增长25%；全市第三产业增加值完成171.9亿元，年均增长10.8%，今年前三季度完成145.4亿元，增长9.7%，预计全年增长9.5%。

  ———坚持抓项目扩投资，基础设施条件明显改善。始终把项目建设作为夯基础、稳增长的强大引擎，加快实施“3341”项目工程，2015年全市固定资产投资完成590.6亿元，较2011年翻了一番，年均增长26.9%；今年前三季度完成448.5亿元，增长11.36%，预计全年完成650亿元，增长10.5%。着力破解交通瓶颈，武罐、成武、十天高速公路建成通车，实现了高速公路零的突破；兰渝铁路陇南段年底建成通车，陇南人的铁路梦将变为现实；陇南成州民用机场有望明年上半年通航，渭武、两徽高速公路全线开工，武九高速公路试验段开工建设，国省干线全面升级改造，建成了洛礼路、南腊路、礼武路等一批县际联网公路，自筹资金建成了五阳路，综合交通网络正在逐步形成。加快新型城镇化建设，市行政中心建成投用，钟楼滩新区、江南小区加快建设，吉石坝新区路网基本形成，长江大道东西延伸一期工程、滨江生态湿地公园及各县城区道路、供排水、污水垃圾处理等一批市政基础设施项目建成投用，两当县被评为全国园林化县城；4个省级新型城镇化、9个小城镇综合改革试点稳步推进，42个乡改为建制镇，全市城镇化率2015年达到28.2%，较2011年提高了6.8个百分点；建成生态文明新农村1870个，占行政村总数的58.4%，创建省级美丽乡村示范村58个，为全省新农村建设创出了路子和经验。世行贷款项目、“8.7”暴洪灾害和“7.22”岷县漳县地震灾后重建项目全面完成。水电能源、城乡电网、信息通讯等基础设施建设快速推进，4G网络实现了城区和195个乡镇全覆盖，重点景区、城区主要公共场所实现了免费WiFi全覆盖，行政村宽带网络覆盖率达到85%。智慧城市建设进展良好，建成陇南市大数据政务中心。

  ———坚持精准施策，扶贫攻坚取得阶段性成效。始终把精准扶贫精准脱贫作为“一号工程”，深入实施“1236”扶贫攻坚行动、双联行动和“1+17”精准扶贫行动，创新精准扶贫方式，倾力打造片区扶贫、产业扶贫、电商扶贫、金融扶贫、旅游扶贫、双联扶贫等扶贫品牌，预计今年年底全市贫困人口下降到39万人，贫困发生率从2011年的53%下降到16%，两当县年底前将实现整县脱贫。全面落实“853”挂图作战要求，紧盯25个特困片区，坚持资金向片区倾斜、项目向片区整合，五年累计投入财政扶贫资金26亿元，争取国开行承诺贫困村社基础设施贷款60亿元，今年已到位20亿元，实施整村推进项目750个，完成易地扶贫搬迁1.6万户7万人，解决了111万农村人口安全饮水问题，完成农村危房改造9.2万户，建成通村硬化路1.2万公里，贫困村动力电实现全覆盖。劳务收入连续三年突破百亿元大关，预计今年输转农村劳动力67万人次，创收131亿元，年均增长13.2%。电商扶贫创出了“陇南模式”，我市被国务院扶贫办命名为全国电商扶贫示范市，荣获2015中国消除贫困创新奖。发展互助资金项目3127个。创新旅游扶贫模式，75个村被列入全国“乡村旅游扶贫重点村”。

  ———坚持绿色发展，生态环境持续改善。紧紧围绕把陇南建成长江上游重要的生态安全屏障，加快实施“两江一水”区域综合治理、生态文明示范市、水生态文明城市建设试点和退耕还林还草、天保工程、长防林建设等重点项目，2015年森林覆盖率达到40.4%，高于全省28个百分点。加快推进“6363”水利保障行动，江河干支流、中小河流治理顺利实施，堤防加固、节水灌溉、安全人饮、水土保持等项目累计完成投资23.5亿元。水、大气污染治理效果明显，我市城市空气质量优良天数比率高于全省平均水平，各县区城市污水处理厂正在加快建设并逐步完善，主要污染物总量减排控制在省定指标以内。矿山、尾矿库综合治理深入推进，尾矿库监测预警应急指挥系统建设进展顺利。积极创建国家级地质灾害综合防治试验区，实施了一批地质灾害治理项目，建成自然灾害监测预警指挥系统和市级水资源实时监控与管理信息系统，新一代天气雷达系统建成投用，防灾减灾能力进一步提升。

  ———坚持改革开放，发展活力不断增强。深化“接放管服”改革，调整取消和下放行政审批事项213项，减幅57.7%；新建了市区两级政务服务大厅，各县区县级政务大厅规范运行，175个乡镇建立了便民服务大厅；“四张清单一张网”建成运行，实现了市县区和重点乡镇行政审批互联互通；“三证合一、一照一码”加快推行，今年年底市场主体将达到11.5万户，较2011年增长94%。工商、质监、卫生计生、食品药品监管等机构改革全面完成。公共资源交易管理和平台建设不断加强。“营改增”试点全面推行，国库集中支付、预算管理改革深入实施。市级公务用车制度改革有序推进。农村土地承包经营权确权登记、集体林权制度改革顺利实施。创新“3+1”金融服务体系，建成全省首家农村产权确权抵押交易服务平台，累计发放贷款50亿元；组建成立了陇南龙江城乡发展有限公司、陇南信通城市经济发展有限公司、陇南龙江金融服务有限公司，农民、中小微企业和项目建设融资难题得到有效缓解。直接融资取得突破性进展，金徽酒业公司在上交所成功上市融资。2015年全市金融机构贷款余额478.6亿元、年均增长24%，预计今年年底贷款余额达到570亿元，存贷比达到70%，较2011年提高了21个百分点。加大招商引资力度，五年累计签约项目796个，到位资金983亿元，年均增长34%。

  ———坚持夯实民生基础，群众生活水平明显提高。始终坚持保基本、兜底线、补短板、惠民生，每年办理一批民生实事，市级财政支出80%以上用于改善民生。农村义务教育营养改善计划全面落实，“全面改薄”、校安工程加快实施，乡镇和2000人以上的行政村幼儿园实现全覆盖，徽县、武都区、成县、两当县顺利通过国家义务教育均衡发展评估认定，学前教育、普通高中和职业教育协调发展，市一中成功创建省级示范性高中，陇南师专升本和陇南护理职业学院创建工作进展顺利，今年高考二本上线率27%，较2011年增长14个百分点。公共卫生服务水平不断提升，农村合作医疗政府补助标准、城镇居民医保报销比例逐年提高，新农合参合率达到98.2%。市“四馆四中心”等一批公共文化体育设施建成投用，乡村舞台加快实施，乡镇综合文化站、农家书屋、广播电视“村村通”“户户通”实现全覆盖。成功举办市第七、第八届运动会。累计完成科技培训73.6万人次，取得科技成果153项。城乡居民社会保障体系不断完善，城乡低保标准、城乡居民基础养老金、退休职工养老金逐年提高。积极推进“大众创业、万众创新”，城镇新增就业成效明显。保障性住房建设、棚户区改造和危房改造进展顺利。安全生产责任和监管措施得到有效落实，全市安全生产形势总体稳定。“六五”普法规划全面完成，“五队一卡”农村社会治理模式全面推行，平安陇南建设进展顺利。民族宗教、外事侨务、统计、审计、档案、地方志、气象、地震、“双拥”、人防、民兵预备役和妇女儿童、残疾人、老龄、慈善、红十字会等各项事业取得了新的成绩。

  ———深入推进转职能改作风，政府自身建设不断加强。全面落实省委、省政府和市委各项决策部署，自觉接受人大法律监督和政协民主监督，积极转变政府职能，行政效能不断提升。坚持科学民主决策，提高依法行政水平，法治政府建设不断加强。严格落实党风廉政建设“3783”主体责任、“866”衡量标尺要求，严格执行中央八项规定、国务院约法三章和省委市委各项规定，全市“三公”经费支出明显下降。认真开展党的群众路线教育实践活动、“三严三实”专题教育和“两学一做”学习教育，持续推进“效能风暴”行动，有效整改“四风”和“不严不实”问题，政府系统服务意识、服务水平不断提升。

  各位代表，过去的五年，是我市综合实力不断增强、改革开放深入推进、城乡面貌持续改善、交通基础设施不断夯实、群众得到实惠更多的五年。五年来，我们始终坚持加快发展这个第一要务，把“四个全面”战略布局和“五大发展”理念与我市实际紧密结合起来，认真贯彻落实省委、省政府和市委各项决策部署，坚持“有所为有所不为，扬长避短、后发赶超”的发展理念，深化实施“433”发展战略，稳增长、促改革、调结构、惠民生，全市经济总量和质量效益大幅提升，2015年全市GDP增幅居全省第一，2014年以来农村居民人均可支配收入连续两年增幅居全省第一，固定资产投资、社会消费品零售总额和财政收入、财政支出较2011年翻了一番。五年来，我们始终坚持改革创新这个第一动力，坚持问题导向，大胆探索创新，着力破解体制机制障碍，电商扶贫、“3+1”金融服务体系、新农村建设、乡村旅游等工作实现了突破性发展，为今后发展创出了可复制、可借鉴、可推广的经验。五年来，我们始终坚持发挥生态这个突出优势，秉持“绿水青山就是金山银山”的发展理念不动摇，坚持保护与开发并重，坚持人与自然和谐相处，生态环境持续改善，退耕还林、农业特色产业、乡村旅游等生态经济发展势头强劲，走出了一条生态良好、产业发展、人与自然和谐的发展之路。五年来，我们始终坚持执政为民这个根本宗旨，把人民对美好生活的向往作为奋斗目标，持续兴办民生实事，尽力解决群众行路难、吃水难、过河难、看病难、上学难等困难和问题，群众生产生活条件得到了极大改善。五年来，我们始终坚持依法行政这个基本保证，严守政治纪律和政治规矩，不断转变政府职能，完善行政决策规则和程序，积极推行政务公开，进一步优化发展环境，认真践行“三严三实”，努力建设法治政府、服务政府，为改革发展稳定提供了坚强保障。五年的成就，充分展现了全市干部群众苦干实干精神和创新创造能力，极大地增强了我们决战脱贫、决胜小康的信心和决心，必将激励我们百倍努力、奋勇前进！

  各位代表，过去五年的成绩来之不易，是省委、省政府正确领导和亲切关怀的结果，是市委统揽全局、科学决策的结果，是市人大、市政协及社会各界监督支持的结果，是各级政府和全市广大干部群众团结一心、共同奋斗的结果。在此，我代表市人民政府，向全市广大干部群众，向省驻陇南各单位、人民解放军和武警驻陇南部队官兵，向各民主党派、工商联、人民团体、无党派人士、离退休老同志，以及所有关心、支持政府工作的社会各界人士表示崇高的敬意和衷心的感谢！

  今后五年的目标和主要任务

  各位代表，今后五年是我市深入实施“十三五”发展规划，决战脱贫、决胜小康的关键阶段。政府工作的指导思想是：高举中国特色社会主义伟大旗帜，全面贯彻党的十八大和十八届三中、四中、五中、六中全会精神，深入学习贯彻习近平总书记系列重要讲话精神，紧紧围绕“五位一体”总体布局和“四个全面”战略布局，积极践行“五大发展”理念，全面落实省委省政府决策部署和市第四次党代会精神，坚持有所为有所不为，扬长避短、后发赶超，深化拓展、深入实施“433”发展战略，坚决打赢脱贫攻坚战，统筹推进经济、政治、文化、社会、生态文明和政府自身建设，加快建设幸福美好新陇南，努力与全省全国一道全面建成小康社会。按照市第四次党代会确定的奋斗目标，全市经济社会发展的目标任务是：生产总值年均增长8%以上，规上工业增加值年均增长8%以上，固定资产投资年均增长10%以上，社会消费品零售总额年均增长9%以上，一般公共预算收入年均增长8%以上，农村居民人均可支配收入和城镇居民人均可支配收入年均增长12%和8%以上，居民消费价格指数、城镇登记失业率、单位生产总值能耗等指标控制在省定目标以内。着力抓好七个方面的重点工作：

  (一)扎实推进精准扶贫，坚决打赢脱贫攻坚战。强化“一号工程”意识，深度聚焦25个特困片区、1365个建档立卡贫困村和39万贫困人口，深入推进“1236”扶贫攻坚行动、双联行动和“1+17”精准扶贫行动，持续增强“六大品牌”的带动效应，确保到2020年实现脱贫攻坚目标。整合项目资金，增加脱贫攻坚专项预算，用足用好金融扶贫政策和贷款，加快贫困村社水、电、路、房等基础设施建设，彻底改善贫困村社基础设施条件。在村村通水泥路的基础上，加快通社公路和便民桥建设，彻底解决群众行路难、过河难的问题；解决798个贫困村、49万人饮水不安全问题，农村安全饮水实现全覆盖；完成危房改造5.2万户，确保农村群众住房安全；完成7.1万人易地扶贫搬迁任务，实现应搬尽搬；加快实施农村宽带补偿试点和“宽带乡村”项目，实现贫困村有线网络全覆盖；文化、教育、卫生等基本公共服务均衡发展。加强财政扶贫资金、惠农资金监管，确保安全有效使用。以贫困群众增收为核心，创新扶贫模式，培育特色富民产业，实施旅游带动、电商驱动、金融支撑、生态补偿、社保兜底等脱贫工程。坚持既要“富口袋”、更要“醒脑袋”，实施精准培训，实现贫困户有需求劳动力培训全覆盖。抢抓机遇，积极主动做好东西扶贫协作工作，实施“百企帮千村”扶贫行动计划，充分发挥职能部门和双联等帮扶单位的作用，着力构建专项扶贫、行业扶贫、社会扶贫“三位一体”的大扶贫工作格局，建成全国扶贫开发示范区。

  (二)积极推进供给侧结构性改革，加快产业转型升级。深入贯彻“五大发展”理念，以推进供给侧结构性改革为主线，按照“两带一轴”产业布局，着力构建“一个战略平台、六大产业集群”，培育特色产业升级版。加快现代农业发展步伐，深入实施“365”现代农业发展行动计划，持续推进特色产业提质增效，做大做强核桃、花椒、油橄榄、中药材等主导产业，加快发展畜牧、蔬菜、茶叶、苹果、马铃薯、苗木等区域性优势产业，因地制宜发展食用菌、水产、蜂蜜等地方性特色产品，实现适宜区全覆盖和规模效益最大化。加快实施“十百千万”工程，培育壮大40户骨干龙头企业，积极创建油橄榄全国知名品牌示范区等10个现代农业示范园区，打造20个省级名牌产品。转变农业发展方式，鼓励引导农民以市场需求为导向，加快调整种养结构，促进种养加一体、一二三产业融合发展；积极推进农村土地、林地流转，大力发展适度规模经营，不断提高农业综合生产能力，农产品加工转化率达到55%以上。全面落实科技增粮措施，实现粮食稳定增产。改造提升传统产业，围绕改造技术设备、降低能耗、提高资源综合利用率，实施一批技术改造、精深加工产业链项目，大力发展新型材料、装备制造等战略性新兴产业，加快矿产资源高端开发综合利用，推动铅锌、黄金等资源型产业转型升级；以市场需求为导向，优化产品结构，延伸产业链条，促进绿色有机食品生产加工、建筑建材清洁能源、现代制药医养保健等传统产业从低档产品、单一产品、初级产品向中高档产品、系列产品、高附加值产品和精深加工产品转化。落实中小微企业扶持政策，降低企业成本，不断提升非公经济规模效益。严格落实企业安全生产、环境保护主体责任，坚决避免安全生产和环境污染事故发生。推动园区经济集约发展，按照产城合一、一二三产高度融合的思路，高标准做好开发区规划，加快陇南经济开发区基础设施建设，争创国家级开发区，把各县区经济开发区打造成省级开发区。创新管理运营机制，拓宽投融资渠道，谋划实施一批PPP项目。进一步优化产业布局，最大限度地集约利用土地，配齐生产要素和市场要素，引进一批与生态相适应的绿色环保、产业链延伸项目，引导优势产业聚集发展，促进开发区与新型城镇化融合发展，形成以产促城、以城兴产、产城融合的园区发展格局，努力实现县域经济提档升级。提升现代服务业发展水平，大力发展农产品仓储、冷链物流、第三方物流和智慧物流等产业形态，加快推进武都区吉石坝现代物流园、宕昌县哈达铺现代物流园、陇南经济开发区空港物流园建设，健全物流仓储产业体系。实施全国电商扶贫示范市建设，完善基础设施，健全产业链，建设区域品牌，推进电子商务提质增效。促进文化旅游融合发展，大力发展山水生态、历史文化、红色纪念、乡村休闲、民俗风情等旅游品牌，创新发展农业工业观光、医疗养生保健等旅游新业态，把陇南打造成全国全省知名的生态休闲度假区、历史文化观光区。实施乡村旅游富民工程，完善乡村旅游配套设施，开发建设养生度假、田园风光、传统民俗、景区辐射、古镇村落等多种类型的乡村旅游村镇，真正做到留住乡愁、装扮乡容、开发乡宝、富裕乡民。围绕打造大九寨旅游经济圈，加强与周边地区的交流合作，打造精品旅游线路，重点抓好10个精品景区建设，争创5A级景区1个、4A级景区7个，促进全域旅游发展。

  (三)坚持实施项目带动战略，着力夯实发展基础。今天的项目数量就是明天的经济总量，今天的投资结构就是明天的产业结构。我们必须始终坚持抓项目建设不动摇，不断提高谋划项目、争取项目和实施项目的能力和水平。加快构建综合交通网络，深入实施“6873”交通突破行动，建成渭武高速、两徽高速、武九高速、成州民用机场和陇南综合客运枢纽等重大交通项目；加快项目前期工作，及早开工建设天水至平凉铁路南延线(陇南段)、通用航空机场和重点景区直升机起降点，积极做好阳平关至陇南至九寨沟铁路、成都至兰州铁路(陇南段)、合作至哈达铺至两当至凤县铁路等项目前期论证工作；加快国省干线公路改造和旅游公路、乡村公路建设，打通联网路、断头路，形成西北西南区域性立体交通枢纽。加强水利基础设施建设，根本解决9县区城区居民生活用水和园区生产用水问题。加快推进云屏水库、宋家湾水库、永固峡水库等水利项目前期工作进度，争取早日开工建设。加强农田水利建设，抓好河谷川坝区灌溉工程、山丘区特色农业节水增效工程建设。加快白龙江、白水江、西汉水主要干支流和中小河流治理，建设一批设防标准较高的堤防工程，消除防汛安全隐患。加快能源基础设施建设，严格落实水能资源开发利用管理办法，科学有序开发水能资源。加强农村电网建设，鼓励发展风电、光电、生物质能源等新能源。加快天然气管网、加气站建设，积极推进天然气户用工程，实现9县区城区、重点镇和经济开发区天然气管道供应。加快智慧陇南建设，为经济社会发展提供信息化支撑。

  (四)统筹推进城乡发展，不断提升新型城镇化水平。树立现代城市发展理念，统筹推进城乡规划、产业发展、基础设施建设、基本公共服务和社会治理五大任务，努力形成以工促农、以城带乡、工农互惠、城乡一体的发展格局。优化城市发展布局，完成市区总体规划修编，进一步修订完善和严格执行市区和各县城市控制性规划，加快编制供水、排污、道路、综合管廊、供热、供气等基础设施专项规划，完成104个中心镇总体规划修编，乡村规划实现全覆盖。统筹城市空间、规模、产业三大结构，既要保护和传承厚重的历史文化，又要彰显和融合自然景观、地域特色、民族风情，着力构建市区、县城、小城镇、美丽乡村“四位一体”的城乡体系，努力打造山水园林宜居城市。完善城市服务功能，加快推进东江新区和钟楼滩、吉石坝、江南小区、汉王片区开发，改造提升旧城区，有序推进8县县城旧城区升级改造和新城区开发。重点抓好市区及各县城区集中供水设施建设及水源地保护项目，延伸城乡供水管网，切实提高供水质量；加快雨污分流管网改造和排水防涝设施建设，切实解决好城市积水内涝问题；规划实施一批供热供气、垃圾污水处理设施和管网等配套项目，实现市、县和重点建制镇污水处理和垃圾无害化处理设施全覆盖；科学谋划和抓紧启动实施一批公园广场、道路桥梁、停车场、城市综合管廊和过境快速通道等项目，不断提升城市综合服务功能。统筹推进新型城镇化建设，用足用好棚户区改造、易地扶贫搬迁等政策，加快推进省级新型城镇化试点和小城镇综合改革，抓好城中村改造和配套基础设施建设，提升城镇综合服务功能，培育壮大县域经济，鼓励引导农村人口向城镇转移，全市城镇化率达到35%以上。加快特色小镇和美丽乡村建设，创建省级特色小镇10个、市级特色小镇30个，建成生态文明新农村2300个以上、美丽乡村200个以上。

  (五)牢固树立绿色发展理念，全面推进生态文明建设。始终坚持生态优先价值取向，紧紧围绕构建长江上游重要的生态安全屏障，切实抓好生态文明示范市、水生态文明城市建设试点、“两江一水”区域综合治理和自然保护区、湿地公园、森林公园等生态建设项目，深入推进天然林保护、公益林和长防林建设、环境治理等项目，建成一批生态文明建设示范区和示范点，全市森林覆盖率达到42.5%以上。探索建立开发地区、受益地区与生态保护地区，以及流域上游地区与下游地区之间有效的协商平台和补偿机制，逐步实现森林、草原、湿地、河流、矿区等重点领域和禁止开发区域、重点生态功能区生态补偿全覆盖。整山系、整流域、整片带推进退耕还林还草，调整优化产业结构，大力发展特色经济林果、林下经济、生态放养鸡和中蜂养殖、生态旅游等生态经济，建成秦巴片区绿色生态产业示范区。实行最严格的环境保护制度，加强矿山环境恢复治理，加大尾矿库整治力度，坚决消除病险尾矿库、堆渣场、采空区等生态安全隐患；大力实施工业废水、废气、废渣全面达标排放计划，积极防治城乡污水垃圾、城市大气污染和农业面源污染，大力整治城乡环境，主要污染物总量减排控制在省上目标以内。实施一批滑坡、泥石流等地质灾害防治项目，充分发挥自然灾害监测预警指挥系统的作用，积极做好监测预警、风险调查、工程防御、宣传教育等工作，不断提升防灾减灾能力。

  (六)统筹社会事业发展，切实增强人民群众幸福感。始终坚持教育优先发展战略，实现县域义务教育均衡发展，全面普及学前教育和高中阶段教育，大力发展适用型职业教育，加快发展特殊教育和高等教育，完成陇南师专升本和陇南护理职业学院创建工作。深入推进国家级医养结合试点市、健康陇南建设，加快实施公共卫生服务能力提升工程、计划生育优质服务示范工程，实现医疗卫生与养老服务资源共享，创建全省乡村休闲养老融合发展示范区。实施文化惠民工程、文化遗产“历史再现”工程，加快城乡公共文化基础设施、文化队伍建设，大力发展文化产业，建设特色文化大市。加快发展体育事业，充分利用已建成的各类体育设施，大力开展全民健身和体育活动。积极实施促进科技成果转移转化行动，加强与高校和科研院所的交流合作，创建国家级农业科技示范园。加快建设“双创”示范基地，努力扩大城乡就业，城镇登记失业率控制在4%以内。积极发展社会福利、慈善、红十字会和残疾人事业，推进城乡低保、社会保险、养老服务、救助救济科学化管理，健全社会保障体系。毫不松懈地抓好安全生产、物价监管、农产品质量安全和食品药品安全监管，及时防范和处置各类突发问题。加强和创新社会管理，深入推进平安陇南建设，严厉打击各类违法犯罪活动，有效化解社会矛盾，确保社会大局和谐稳定。

  (七)不断深化改革开放，持续增强发展活力。深入推进行政审批制度改革，抓好“接放管服”改革任务落实，规范政务大厅运行，行政审批事项进驻率达到100%。持续深化商事制度改革，实现“五证合一、一照一码”，加快推行电子营业执照。完成农村土地承包经营权、宅基地所有权、集体林权确权登记颁证工作，充分发挥农村产权确权抵押交易服务平台的作用，实现农村产权市场化配置。持续推进财税体制改革，全面落实“营改增”试点和结构性减税政策。加快推进医疗卫生、药品供应和监管体制综合改革。加快供销社改革，完成国资国企、户籍制度改革任务。推进投融资体制改革，推广运用“PPP”等项目建设运营模式，提升投融资平台运作水平。深化金融改革，持续推进“3+1”金融体系建设，不断扩大社会融资规模。加大招商引资力度，加强与川渝、陕南等毗邻地区在文化旅游、特色产业开发、资源利用、生态保护、基础设施等领域的务实合作，加快构建全方位、多层次的开放新格局。

  2017年的主要工作任务

  2017年是新一届政府的开局之年，做好2017年的政府工作，对新一届政府起好步、开好局，意义重大、影响深远。全市经济社会发展的主要预期目标是：生产总值增长8%，规上工业增加值增长8%，固定资产投资增长10%，社会消费品零售总额增长9%，一般公共预算收入同口径增长8%，城镇居民人均可支配收入增长8%以上，农村居民人均可支配收入增长12%以上，城镇登记失业率控制在4%以内，人口自然增长率控制在8.9‰以内，居民消费价格指数控制在3%以内，单位生产总值能耗、污染减排完成省上下达的年度任务。2017年的具体工作任务，我们将根据全国、全省经济工作会议精神，召开全市经济工作会和市政府全体会议进行全面安排部署，重点抓好八个方面的工作：

  一是全面完成年度脱贫任务。实现徽县、成县整县脱贫，10万人稳定脱贫。建成村社道路200公里，解决18万人饮水不安全问题，改造农村危房2万户，完成易地扶贫搬迁1.5万人。深入开展特色产业提质增效活动，完成经济林综合管理650万亩，中药材标准化种植面积达到42万亩，发展生态放养鸡520万只、中蜂养殖20万箱，新建标准化养殖场(小区)30个，确保贫困户人均收入增速高于全市平均水平。加快创建陇南市国家级现代农业科技示范园、礼县国家级出口食品(苹果)质量安全示范区、宕昌县国家中医药产业发展综合示范区。培育壮大核桃、花椒、油橄榄、中药材、苹果等特色农产品精深加工企业，推动“企业+基地+农户”一体化生产。积极培育发展农民合作社、家庭农场、专业大户等经营主体，健全带贫机制，实现群众持续增收。加强耕地保护，全面落实科技增粮措施，确保粮食稳产增收。进一步加大劳动技能培训力度，实现向技能型、有组织长期稳定输出转变，不断拓宽群众增收渠道。

  二是加快推进项目建设。陇南成州民用机场建成通航，渭武、两徽高速公路完成年度投资计划，武九高速公路加快建设，力争全线开工；加快天水至平凉铁路南延线(陇南段)前期工作进度。实施一批国省干线升级改造、旅游公路和乡村公路建设项目。启动县区城市供水工程，完成规划河道污染物应急控截工程，开展主要干支流、中小河流、山洪沟道治理和各县区城区防洪沟道治理，实施滑坡、泥石流等地质灾害防治项目，全面完成退耕还林还草、天保工程、公益林等生态文明建设任务。加快电网建设，确保330千伏晒金变至礼县变二回线路，110千伏西和变、文县变增容等项目建成投用。加快推进宽带网络覆盖工程，行政村通宽带率达到95%以上。

  三是强化工业转型升级。落实直购电、电价补贴等扶持政策，争取将铅锌、黄金采选冶和水泥企业纳入直供电范围。实施市列重点工业项目20个，金徽酒业优质酒技改、郭家沟铅锌矿采选、礼县青峰石材扩建等项目实现达产达标，力争成州锌冶炼废渣综合利用、礼县鑫晟源大黄精深加工二期、中宝矿业小东沟黄金采选、西和县青羊矿业公司铅锌技改等项目建成投产，加快厂坝公司300万吨扩能、紫金矿业尾渣综合利用二期工程及黄金冶炼厂等项目前期工作进度，力争尽快开工建设，新增规上工业企业5户以上。加快推进陇南经济开发区扩区增容，明确功能定位和核心区，完成控制性详规修编工作。采用PPP等多种形式统筹推进开发区道路桥梁、垃圾污水处理和供水、供电、供气等基础设施建设，全面拉开陇南经济开发区基础设施建设框架。整体谋划、分区建设、统筹推进各县区经济开发区建设。依托资源和区位优势，积极开展园区招商，力争在引进企业、壮大产业上取得实质性进展。

  四是大力发展现代服务业。完善武都区吉石坝现代物流园、宕昌县哈达铺现代物流园基础设施，启动陇南经济开发区空港物流园、大堡子山国家考古遗址公园、文县白马人原生态文化旅游区、阳坝文化自然遗产保护和国家湿地公园等文化旅游重点项目。抓好宕昌官鹅沟5A级景区及文县天池、成县鸡峰山、徽县金徽矿业、宕昌县哈达铺等4A级旅游景区创建工作。发挥“十村百户千床”工程的示范作用，新建一批乡村旅游扶贫村。抓好电子商务进农村示范县建设。加强历史文化名镇名村和传统村落保护开发，抓好“乞巧节”、“蜀道”、“北茶马古道”申报世界文化遗产工作。全市旅游综合收入增长20%、文化产业增加值增长25%以上。

  五是统筹推进新型城镇化。修订完善市区、8县县城和市县区园区规划，延伸市区东江新区基础设施配套，开展汉王片区土地统征储备和相关项目建设；谋划实施一批公园广场、停车场等公共服务设施，进一步完善老城区服务功能。积极争取国家专项建设债券基金，协调解决影响项目建设的困难和问题，加快实施长江大道东西延伸二期工程、东江新区外环路、阶州广场、南山公园、阶州植物园和八县县城道路、公园、停车场等市政基础设施建设项目；开展阶州古街、滨江体育场、南长江大道和市县区城市综合管廊、过境快速通道等项目前期工作。完成市区和各县城区供水基础设施项目前期论证工作；抓好东江新区污水处理厂搬迁改造、吉石坝污水处理厂建设，加快完善各县城区污水处理厂基础配套，加大收集管网建设力度，稳步提高污水收集率和处理率；抓好城乡垃圾处理项目建设，进一步完善垃圾收运和中转系统，逐步实现垃圾减量化、资源化、无害化；抓好市区和有供热项目县的集中供热项目建设，积极稳妥扩大供热面积，稳步提高供热企业效益。抓好棚户区改造、危房改造建设项目，推动房地产市场健康平稳发展。统筹推进康县、礼县、成县小川镇、宕昌县哈达铺镇等省级新型城镇化试点和9个小城镇综合改革试点，加快基础设施和公共服务设施、产业发展项目建设，持续抓好户籍、土地、投融资等改革。加快特色小镇建设，实施高速公路沿线村镇风貌改造工程。加强城市综合管理，有效解决城区和重点镇交通堵、停车难、秩序乱、环境差等“城市病”。扎实开展农村人居环境改善行动，建设一批生态文明新农村和美丽乡村。

  六是全力做好财税金融工作。充分发挥财源建设基金的作用，培育壮大规上企业，加大对新兴产业、中小微企业的扶持力度，大力发展现代服务业、商贸物流业，优化财源结构，积极培植新的增长点。加强对有色金属采选业、建筑业、房地产业等重点税源的监控，加大各类工程项目建设、资金拨付进度，实现财税收入稳定增长。全面推进“营改增”扩围改革，加强小税种征管，加大欠税清缴力度，严格落实非税收入管控措施，确保足额入库、应收尽收。进一步优化支出结构，把保运转、保民生、保稳定、促发展摆在突出位置，加快一般公共服务、教育、社会保障和就业、医疗卫生等重点民生领域的支出进度，确保财政八项支出持续增长。及时清理财政存量资金，加强对项目建设、资金使用情况的跟踪督查，加快支出进度，提高资金使用效率。深入开展“小金库”专项治理，切实加强财政资金监管。全面落实“双联”惠农贷款、精准扶贫贷款、易地扶贫搬迁和草食畜牧业、设施蔬菜、中药材产业等贴息贷款政策，扩大惠农贷款覆盖面，为农业特色产业发展提供资金保障。不断提升农村产权确权抵押交易服务平台的运营水平，健全配套机制，实现市县区和重点乡镇联网运行。进一步规范政府投融资公司管理体制，充分发挥陇南龙江金融服务公司和产业发展基金的作用，通过市场化运作、封闭式运行和透明化管理，实现银企、银农合作，破解融资难题。发挥市信通公司的职能作用，加快推进官鹅沟自驾游房车营地、客运索道和康县长坝温泉度假村、古道民俗集市等项目建设。推动各金融机构加大信贷投放力度，确保全年信贷投放增速积极稳定增长。

  七是加快发展社会事业。推进县域义务教育均衡发展，抓好“全面改薄”、行政村幼儿园改扩建项目，陇南师专升本和陇南护理职业学院创建取得实质性进展。推进公共卫生服务均等化，健全医保体系，改善乡村医疗卫生条件，实施万名医生支援农村卫生工程，提升医疗卫生服务水平。抓好社区综合文化服务中心、街道综合文化站建设，实现“乡村舞台”全覆盖。建设养老机构、城乡社区老年人日间照料中心等项目。充分发挥康县花桥、徽县泥阳农民工返乡创业基地和陇南电子商务产业孵化园创业基地的示范作用，新增城镇就业2.8万人。落实好城乡低保、特困人员供养、医疗救助等政策。抓好民族宗教、红十字会和慈善、双拥、民兵预备役、妇女儿童、老龄、残疾人等各项工作。2017年，市政府将多渠道筹集资金，重点围绕脱贫攻坚和社会保障、就业创业、就医上学、住房交通、环境保护等民生问题，继续办好民生实事，全力抓好省列为民办实事项目，积极办理人大代表建议意见和政协委员提案，切实解决好群众最现实、最关心、最直接的利益问题。

  八是抓好安全生产和维稳工作。严格落实安全生产监管责任和各项监管措施，加强重点领域和重点行业隐患排查整治，严防各类安全生产事故发生。紧盯尾矿库“一库一策”治理方案，抓好应急预案演练、尾矿库监测预警应急指挥系统建设、“头顶库”群众搬迁等工作。持续开展食品药品安全专项整治行动，加强农产品和食品药品安全监管。抓好群众信访和矛盾纠纷排查调处工作，加强基层社会管理和服务体系建设，创新社会治理体制，不断提高基层治理水平。深入推进“七五”普法工作，加快推进依法治市进程。深化“平安陇南”建设，努力营造和谐稳定的社会环境。

  各位代表，实现未来五年的宏伟目标，完成明年的各项任务，对政府工作提出了新的更高要求，我们必须坚持不懈地加强政府自身建设。一是推进依法行政，建设法治型政府。健全行政决策机制，完善重大事项集体决策、专家咨询、社会公示与听证制度，提高政府决策的科学化、民主化、规范化水平。规范“四张清单一张网”运行，推行市场准入负面清单，加强行政权力运行监督。自觉接受人大及其常委会的法律监督、工作监督和政协的民主监督，提高依法行政水平。二是提高行政效能，建设服务型政府。继续简政放权，对直接面向基层、量大面宽、由县区实施更方便更有效的行政审批事项，一律下放县区实施。做好已取消下放行政审批事项的落实和衔接工作，为大众创业、万众创新营造良好环境。大力推行电子政务和网上审批、网上办事，全面深化政务公开，规范政务大厅运行，实现与国家、省级平台互联互通，为群众提供便捷高效服务。三是改进工作作风，建设务实型政府。紧盯目标任务，凝心聚力谋发展，聚精会神抓落实，确保政令畅通、执行有力。不断加强政府系统能力建设，坚持用制度管人管事，培养一支政治坚定、业务精湛、作风过硬、群众满意的公务员队伍。进一步健全目标责任管理和绩效考核机制，对重点工作、重大项目和民生实事，明确时间表和路线图，及时督查考核，跟踪问效，严格兑现奖惩，做到令必行、行必果。严格执行中央“八项规定”、国务院“约法三章”和省委“双十条规定”、市委“十二条规定”，持之以恒整治“四风”，认真践行“三严三实”，继续深入开展“两学一做”学习教育，不断提升政府系统服务效能。四是坚持廉洁从政，建设廉洁型政府。认真落实政府系统党风廉政建设主体责任和领导干部廉洁自律各项规定，全面落实省委“3783”主体责任、“866”衡量标尺、“111”督查落实细则和市委“663”工作机制要求，建立健全重点领域廉政风险防控机制，强化行政监察和审计监督，规范土地开发、政府采购、项目资金安排、国有资产转让、重大工程建设招投标管理，严肃查处违纪违法案件，坚决纠正损害群众利益的不正之风。大兴勤俭节约、艰苦奋斗之风，压减“三公”经费支出，树立为民务实清廉的良好形象。

  各位代表，实现市第四次党代会确定的各项目标，是全市人民的共同期待，也是新一届政府的神圣使命和重要职责。让我们在省委、省政府和市委的坚强领导下，在市人大和市政协的监督支持下，团结带领全市人民，开拓创新，真抓实干，敢于担当，主动作为，努力完成本届政府各项目标任务，为坚决打赢脱贫攻坚战，与全省全国一道全面建成小康社会而努力奋斗！
